# Outcomes in patients who received ECMO and/or volatile anesthetics as rescue therapies for status asthmaticus★

**DOI:** 10.1051/ject/2024008

**Published:** 2024-09-20

**Authors:** Kavipriya Komeswaran, Deanna Todd Tzanetos, Tiffany Wright, Jamie Furlong Dillard

**Affiliations:** 1 University of Mississippi Medical Center Jackson MS USA; 2 University of Louisville Louisville KY USA

**Keywords:** ECMO, Status asthmaticus, Inhaled volatile anesthetics, Pediatrics

## Abstract

*Background*: In the state of Kentucky, many status asthmaticus (SA) patients require care in the Pediatric Intensive Care Unit (PICU) and a fraction of these patients may receive “rescue therapies” with inhaled volatile anesthetics (IVA) and/or Extracorporeal Membrane Oxygenation (ECMO). We present a series of such patients with the objective of comparing the clinical parameters of individual patients who received inhaled volatile anesthesia and subsequently the need for ECMO. *Methods*: Children between 2 and 18 years of age admitted to our PICU from January 2014 to July 2020 with SA were reviewed and categorized as 1) patients who received IVA alone, 2) patients who received IVA and then subsequently ECMO, and 3) patients on ECMO alone. *Results*: A total of 1772 children with SA episodes were identified with a mortality of 13 patients. Seven children with SA were identified who received either IVA, ECMO, or both. One patient received only IVA, 5 received both IVA and ECMO and one received only ECMO. All received standard asthma therapies of steroids, albuterol, magnesium sulphate, and aminophylline prior to escalation. Six out of seven refractory SA received IVA, and five (83%) of those were subsequently escalated to ECMO. There was an improvement in mean pH after cannulation compared to IVA. pCO_2_ levels had no improvement after IVA administration but decreased by an average of 20 points after ECMO. Patients peak inspiratory pressures decreased within the 1st 24 h of ECMO cannulation from a mean of 30 to 18. There were no other complications related to ECMO placement. *Conclusion*: While we cannot decisively draw any conclusions from our study due to the small sample, it was noted that there was no clear advantage of using IVA prior to ECMO in our patients. Most patients who received IVA were escalated to ECMO indicating that early ECMO cannulation may be beneficial. Given the high cost and potential complications of both, there is a need for the development of well-defined guidelines for severe SA management in the PICU.

## Introduction

Status asthmaticus (SA), defined as persistent wheezing and respiratory distress that fails to respond to conventional medical therapy and leads to respiratory failure, is one of the most common indications for admission to the PICU [1]. In Kentucky, particularly counties that fall in the “Ohio Valley Asthma Belt” are known to be notorious for higher asthma rates in children and with increased severity [2]. In 2018, Kentucky had an pediatric asthma prevalence rate of 9.4% (compared to national average of 9%) with mortality rates from asthma of 8.7/million (compared to a national average of 11.3/million) [3].

Although children with respiratory failure secondary to SA predominantly respond to a variety of non-invasive therapies including continuous nebulized beta-adrenergic agonists, corticosteroids, magnesium sulfate, methylxanthines, and noninvasive ventilation, 2–20% of those admitted to the PICU still require intubation and mechanical ventilation [4–6]. Practice patterns for the treatment of SA vary and there are no published guidelines on the treatment of asthmatics sick enough to require the ICU [7]. The definition of refractory SA and decision to continue the escalation of care is within itself subjective and often secondary to the provider’s individual decisions or patient’s side effects from medical interventions. This leaves us with a lack of data on the best treatment modalities for patients who “fail” standard asthma therapies. When these patients further decompensate, despite aggressive methods, they may receive “rescue therapies” with unknown comparative effectiveness, such as inhaled volatile anesthetics (IVA) and/or Extracorporeal Membrane Oxygenation (ECMO) [8].

Recent literature from the Extracorporeal Life Support Organization (ELSO) registry has reported among the small population that receive ECMO for SA, survival rates are as high as 94% [8, 9]. There is even less reported data describing the benefit and appropriate timing when using IVA as a rescue therapy for SA [10–12]. While ECMO and IVA are both considered “rescue therapy”, it is unclear from existing literature if the use of IVA prevents the further need for ECMO support and if there is a comparative difference in mortality between the two therapies.

In our study, we look at a series of patients with status asthmaticus who received either IVA, ECMO, or both as rescue therapy after failing conventional bronchodilator therapies. Our aim was to compare clinical parameters of individual patients who received IVA and subsequently ECMO as rescue therapies and to describe the morbidities and mortality rates among patients who received these therapies in our institution. Our study will add to the small existing literature on use of unconventional rescue therapies for SA and specifically report outcomes on patients who received IVA prior to ECMO.

## Materials and methods

### Study design

This is a retrospective descriptive study of children between 2 and 18 years of age, seen at an urban academic tertiary care Children’s Hospital PICU. Using a combination of the Virtual Pediatric Systems (VPS) database and our institution’s Extracorporeal Life Support Organization (ELSO) data, patients who were admitted to the PICU between January 2014 and July 2020 with an ICD 10 code of SA who received IVA and or ECMO were identified. A retrospective chart review was done which identified a total of 1772 children with status asthmaticus admitted during this time period. Patients with status asthmaticus who received IVA and or ECMO were included in our study. A total of seven patients who received these rescue therapies were identified. These patients were then categorized into three cohorts: 1) patients who received IVA alone, 2) patients who first received IVA and then subsequently received ECMO, and 3) patients who received ECMO alone. The need for informed consent was waived, and institutional review board approval was obtained. ECMO circuits used in our unit had a Rotaflow pump and were primed with blood. Anesthesia ventilators to administer volatile anesthetics were co-managed with the help of anesthesiologists. Sevoflurane and Isoflurane were utilized at a starting dose of 0.5% and titrated to achieve the desired therapeutic effect. Patient data collected included demographic characteristics, therapeutic interventions before rescue therapy, ventilatory parameters, ICU and hospital length of stay, days on ECMO, ventilator days, number of days on sedation, and oxygen support complications, and mortality. The initial and subsequent blood gas parameters and ventilatory data chosen were PEEP, mean airway pressure (MAP), peak inspiratory pressure (PIP), pH, PaO_2_, and PaCO_2_. The initial lab and ventilatory data were the closest reported documentation before IVA or ECMO was initiated. The subsequent labs and ventilatory data were within 4 h after the initiation of rescue therapy.

### Statistics

The identified cohort was small and is presented as a comparison among invasive rescue therapies, but the small sample size precluded meaningful statistical comparisons between all groups. When appropriate, data were described using median values with 25th and 75th interquartile or as percentages.

## Results

A total of 1772 children with status asthmaticus were admitted to our PICU during the time frame of our study. Seven children with SA were included, who received only IVA (*n* = 1), IVA and then ECMO (*n* = 5) or ECMO alone (*n* = 1). Demographic and clinical features are compared between the three groups in Table 1. All children were in the young childhood range (2–8 years). Five of the 6 patients were males. All children received standard asthma therapies of steroids, albuterol, magnesium sulphate and aminophylline prior to escalation with similar dosing ranges for all therapies. There were no established criteria which determined how patients who did not respond to conventional therapies were escalated. The patient on IVA alone received it for 166 h and the mean hours on IVA was much less (50 h) for those who went on to receive ECMO with all 5 patients coming off IVA once cannulated. The mean hours on ECMO were 168 among those who received IVA prior and much longer (456 h) than the patient who received ECMO alone. This specific patient was complex with a history of prematurity and developed acute respiratory distress syndrome after initial presentation for SA.

**Table 1:** Demographic and clinical features of patient cohorts receiving ECMO, IVA + ECMO, and IVA.

	Volatile anesthetic agent alone (*n* = 1)	Volatile anesthetic agent followed by ECMO (*n* = 5)	ECMO alone (*n* = 1)
Age (years) (median, IQR)	4	5 (2, 8)	4
Weight (kg) (median, IQR)	22	17.9 (11.2, 35.0)	12.8
Males (*n*, %)	1	4 (80)	0
PICU asthma medications (*n*, %)			
Methylprednisolone^a^	1	5 (100)	1
Inhaled Albuterol^b^	1	5 (100)	1
Magnesium sulphate^c^	1	5 (100)	1
Aminophylline^d^	1	5 (100)	1
Terbutaline^e^	1	5 (100)	0
Mechanical ventilation days (median, IQR)	14	14.35 (11, 38)	42
Hospital length of stay (median, IQR)	38	37 (13, 78)	78

a Methylprednisolone 1 mg/kg q6h or max dosing of 80 mg BID IV.

b Albuterol dosing for all patients weight based (5–15 mg) as a continuous inhaled infusion or q2h.

c Magnesium sulfate dosing titrated for goal Magnesium level 4–6 mg/dL (range 25–30 mg/kg/h).

d Aminophylline drip with dosing range 0.5–1 mg/kg/h, 2 patients received a bolus of 6 mg/kg prior to drip.

e Terbutaline dosing 1–2 mcg/kg/min.

Table 2 describes each of the five patients who received inhaled anesthesia and then subsequently ECMO. Four of the five ECMO patients were cannulated percutaneously onto venovenous (VV) ECMO, which is our institution’s practice for ECMO cannulation when possible. One patient went on VV initially and one was converted to venoarterial (VA) secondary due to cardiac tamponade on ECMO. This was the only patient who died secondary to neurological injury during this event and withdrawal of ECMO support due to poor neurological prognosis. The patient who was placed on VA initially despite IVA had worsening pCO_2_ to almost 250 mm Hg, and ECHO revealed significant right-sided cardiac failure from pulmonary hypertension caused by hyperinflation and therefore VA was chosen. Timing of ECMO initiation, length of IVA use, and hours of mechanical ventilation were all variable among each patient. One patient on ECMO was found to have developed an occipital subdural hematoma, noted two days after decannulation which did not require any neurosurgical intervention with spontaneous resolution on repeat imaging. One patient on VA ECMO had a large acute non-hemorrhagic infarct of left middle and posterior cerebral arteries found on post ECMO imaging as well, which did lead to right-sided hemiplegia.

**Table 2:** Comparison of clinical parameters of patients who received inhaled volatile anesthesia and subsequently ECMO as rescue therapy for status asthmaticus.

Patient	Total ECMO run time (hours)	ECMO type	Ventilator hours prior to ECMO initiation	Hours on IVA before ECMO use	Ventilator hours after ECMO until extubation	Complications	Mortality
1	176	VV	128	1.5	937	Occipital hematoma	N
2	48	VV	4	0.25	48	n/a	N
3	168	VV	88	46	–	n/a	N
4	192	VV > VA*	120	120	720	Mortality	Y
5	144	VA	72	54	96	Infarct	N

* Patient initially started on VV ECMO and converted to VA ECMO. Abbreviations: VV – venovenous; VA – venoarterial.

Table 3 describes patient’s blood gas and ventilator data at multiple time points. Patients had a mean initial pH of 7.08 with an improvement to an average of 7.17 after IVA and improvement to 7.32 after cannulation. PaCO_2_ levels had no consistent notable improvement in hypercarbia after IVA administration but were found to decrease by an average of 20 points after ECMO cannulation. All patients who were escalated from IVA to ECMO (#1–5) had persistent bronchospasm despite IVA, and 4 of the 5 had persistent acidosis. The timing of the decision was left to the decision of the medical team, and it is unknown if patients had toxicity from IVA contributing to the decision for ECMO. The MAP decreased similarly on IVA and ECMO (from 19 to 13 and then to 12). Patients PIP decreased after IVA by only 2 but within the first 24 h of ECMO cannulation by 10 (from a mean of 36 to 26).

**Table 3:** Change in pH, CO_2_, mean airway pressure (MAP) and peak inspiratory pressure (PIP) prior to rescue therapy (IVA and/or ECMO) and after.

	pH			CO_2_			MAP			Pip		
Patient	Pre-rescue therapy	First gas after Initiation of IVA	First gas after ECMO cannulation	Pre-rescue therapy	First gas after initiation of IVA	First gas after ECMO cannulation	Pre-rescue therapy	On IVA	On ECMO	Pre-rescue therapy	On IVA	On ECMO
1	7.27	7.34	7.46	81	90	58	20	17	13	45	45	32
2	6.97	6.93	7.09	114	110	78	31	14	12	60	50	33
3	7.01	7.13	7.48	102	125	56	18	11	13	18	32	28
4	7.0	7.0	7.25	125	60	83	11	11	11	30	22	16
5	7.08	7.24	7.27	99	65	86	21	14	12	30	28	24
6	7.03	7.21	n/a	103	183	n/a	7	14	n/a	32	30	n/a
7	7.28	n/a	7.42	79	n/a	66	26	n/a	14	40	n/a	27

## Discussion

The use of IVA as a “rescue therapy” for SA is utilized in our institution. Six out of 7 patients with SA refractory to standard medical therapy, received IVA, however, 5 (83%) of those were subsequently escalated to ECMO. Although our case number is small, the sequence of rescue therapy would indicate that there is not a clear advantage to using IVA prior to ECMO in severe SA.

The use of ECMO overall, including for SA, is increasing [13]. Reports from ELSO from 1986 to 2007 indicate a significant increase in the use of ECMO for pediatric respiratory failure and 66% of patients cannulated for SA specifically occurred after 2002. This trend is coinciding with an increased use of VV ECMO [14]. In 2015, 47% of pediatric ECMO was utilized for respiratory failure with the best survival to hospital discharge in patients with SA (92% survival) [15, 16]. Our institution’s experience was in line with this trend as 86% of patients with severe SA refractory to standard medical therapy required ECMO cannulation and 83% of those patients survived.

Reported data on the use of IVA for SA is even more limited and has not shown clear benefit over ECMO as a rescue therapy for this population. IVAs are known to be potent bronchodilators and are effective in improving oxygenation, lowering CO_2_ levels and possibly decreasing ventilator-induced lung injury (VILI) in SA [17]. The CO_2_ and ventilatory parameters that would contribute to VILI in our patient population were more markedly improved after ECMO cannulation. This was likely secondary to sweep gas initiation and change in ventilatory settings based on the common method of “rest settings” and not necessarily a function of alleviation of bronchospasm. Therefore, true beneficial conclusions cannot be made [18]. We also do not have data on plateau pressures which would indicate true alveolar harm in this physiology. It is also important to take caution to not decrease the CO_2_ in severe hypercarbia rapidly to avoid rapid changes in cerebral blood flow [19]. The debatable question that arises is not which rescue therapy is most beneficial, but which is less harmful.

In one of the largest single-center studies conducted by Hebbar et al on IVA use in patients with SA, 8 out of 13 patients who received IVA were still escalated to ECMO. The patients who received both IVA and ECMO as rescue therapies had longer hospital LOS, longer ventilator hours after decannulation, and more hospital charges compared to those on IVA alone [16]. The median time on ECMO was only 95 h. In the larger ELSO SA patient population, the median hours on ECMO were even less (91 h) [16].While both ECMO and IVA have significant side effect risks, the most concerning ECMO risks are all decreased with VV cannulation [20].Although our median time on ECMO in the patients who received both IVA and ECMO was longer at 168 h, the combined benefit of VV > VA ECMO plus short run times would indicate ECMO is a beneficial rescue therapy for refractory SA. In addition, it is worth noting that in our institution, all patients are cannulated percutaneously, further reducing complications related to cut-down cannulation technique. Comparing these studies directly is difficult but the trends could indicate that patients who are sick enough to receive IVA and then ECMO have increased ventilator days and hours on ECMO due to severity of illness. It is also plausible that delaying ECMO and using IVA first could have contributed to increased patient morbidities.

The risks with IVA, while less studied, include profound hypotension caused by a drop in systemic vascular resistance, nephrotoxicity, carbon monoxide toxicity, and cognitive deficits [21, 22]. Perhaps the most important risk from IVA is the risk of long-term neurotoxicity, particularly in the developing brains of children who would be exposed to much longer durations of inhaled anesthetics than typical of an operating room procedure [23, 24]. While the neurodevelopmental impact of early exposure to general anesthesia (GA) in the pediatric population is still poorly understood, in vitro and in vivo studies have consistently shown that exposure produces dose-dependent and developmental age-dependent effects on various neuronal transmission systems [25]. The Food and Drug Administration warning for risk of neurodevelopmental effects due to anesthesia is for > 3 h [26]. There is an increased risk for neurodevelopmental deficits in young children (<4 years) especially [27, 28]. The median age in our cohort was 5 years with ranges from 2 to 8 years, a stage of developmental vulnerability. The median hours on IVA were 50 h which puts them at significantly increased risk of neurodevelopmental side effects. Neurodevelopmental outcomes after ECMO use in patients are as low as 4% in VV patients and correlated with time spent on ECMO [29].

Based on this cohort of patients and known literature on rescue therapy for SA it is not possible to say there is a clear advantage of one over another, however, it is possible that the risk of side effects is lower in patients who are candidates for VV ECMO [30].The data on increased neurological side effects and neurodevelopmental outcomes from increased exposure to IVA is concerning and should be considered when making decisions on rescue therapy in SA. This is especially a concern when it seems most patients who are sick enough to receive IVA are escalated to ECMO regardless based on our cohort as well as prior publications. Lastly, it should be mentioned that the definition of refractory SA requiring rescue therapy is also not clearly defined. Reports show that hypercarbia as high as 500 is shown to not cause harm [31, 32]. The decision to utilize either rescue therapy discussed here is subjective in nature and often guided by toxicity to the medical interventions or physician anecdotal experience. Despite elevation in CO_2_, often the pH is what guides next steps in rescue therapy and our initial mean pH was 7.08.

This review is limited in its conclusions due to the retrospective nature of the data collection and small sample size. We also were not able to identify the denominator of patients who met the definition of SA receiving standard medical therapy in the ICU to know the exact prevalence of the need for rescue therapy at our institution. Also due to the retrospective nature, timing of blood gases and ventilator data were not standardized, and long-term follow-up and neurodevelopmental outcomes are unknown. Minimal data were missing in these five patients presented. It was also not possible to know the medical decision tree framework of each intensivist when rescue therapy was chosen or when the patient was escalated from IVA to ECMO without a clear guideline or standardized method for escalation to rescue therapy at our institution.

## Conclusion

The use of ECMO and IVA for patients with SA varies significantly among institutions based on medical team variation in decision. While we cannot decisively draw any conclusions from our small study, there does not seem to be a clear advantage of using IVA as therapy prior to escalation of this patient population to ECMO. Given the high cost and potential complications of both rescue therapies, there is a need for more prospective trials and development of well-defined guidelines for severe SA management in the pediatric ICU setting.

## Data Availability

All available data are incorporated into the article.
